# Associations of *MAP2K3* Gene Variants With Superior Memory in SuperAgers

**DOI:** 10.3389/fnagi.2018.00155

**Published:** 2018-05-29

**Authors:** Matthew J. Huentelman, Ignazio S. Piras, Ashley L. Siniard, Matthew D. De Both, Ryan F. Richholt, Chris D. Balak, Pouya Jamshidi, Eileen H. Bigio, Sandra Weintraub, Emmaleigh T. Loyer, M.-Marsel Mesulam, Changiz Geula, Emily J. Rogalski

**Affiliations:** ^1^Neurogenomics Division, Translational Genomics Research Institute, Phoenix, AZ, United States; ^2^Cognitive Neurology & Alzheimer’s Disease Center, Northwestern University Feinberg School of Medicine (NU FSM), Chicago, IL, United States; ^3^Department of Pathology, Northwestern University Feinberg School of Medicine (NU FSM), Chicago, IL, United States; ^4^Department of Psychiatry and Behavioral Sciences, Northwestern University Feinberg School of Medicine (NU FSM), Chicago, IL, United States; ^5^Department of Neurology, Northwestern University Feinberg School of Medicine (NU FSM), Chicago, IL, United States

**Keywords:** aging, Alzheimer’s disease (AD), Alzheimer’s dementia, Alzheimer’s, successful aging, genetics, whole exome sequencing, cognition, episodic memory

## Abstract

**Introduction**: SuperAgers are adults age 80+ with episodic memory performance that is *at least* as good as that of average middle-aged adults. Understanding the biological determinants of SuperAging may have relevance to preventing age-related cognitive decline and dementia. This study aimed to identify associations between genetic variations and the SuperAging phenotype using Whole Exome Sequencing (WES).

**Methods**: Sequence Kernel Association Combined (SKAT-C) test was conducted at the gene level including both rare and common variants in 56 SuperAgers and 22 cognitively-average controls from the Alzheimer’s disease Neuroimaging Initiative (ADNI).

**Results**: The SuperAging phenotype was associated with variants in the Mitogen-Activated Protein Kinase Kinase 3 *(MAP2K3)* gene. Three single nucleotide polymorphisms (SNPs) contributed to the significance (rs2363221 [intron 1], rs2230435 [exon 5], rs736103 [intron 7]).

**Conclusions**: MAP2K3 resides in a biological pathway linked to memory. It is in a signaling cascade associated with beta-amyloid mediated apoptosis and has enriched expression in microglia. This preliminary work suggests MAP2K3 may represent a novel therapeutic target for age-related memory decline and perhaps Alzheimer’s disease (AD).

## Introduction

Average episodic memory capacity is significantly higher in populations of 50–60 year olds than in populations of 80+ year olds. We defined SuperAgers as individuals 80 or older with episodic memory at least average for cognitively average individuals in their 50 s and 60 s. Thus, SuperAgers have superior memory capacity for age and are potentially resistant to age-related decline (Harrison et al., 2012; Rogalski et al., 2013; Gefen et al., 2015; Cook et al., 2017). Previous work has shown that SuperAgers are less vulnerable to age-related cortical atrophy (Harrison et al., 2012; Rogalski et al., 2013; Cook et al., 2017), and have thicker anterior cingulate cortex when compared to their “cognitively average” peers (Harrison et al., 2012; Rogalski et al., 2013). Postmortem studies showed more von Economo neurons and less Alzheimer pathology in the anterior cingulate of SuperAgers compared to their peers (Rogalski et al., 2013; Gefen et al., 2015). Here we investigated the association between genetic variation and the SuperAging phenotype using Whole Exome Sequencing (WES).

## Materials and Methods

Analyses included 56 SuperAgers from the Northwestern SuperAging Program. Genotypes were obtained from 22 cognitively-average controls from the Alzheimer’s disease neuroimaging initiative (ADNI) Whole Genome Sequencing (WGS) database^1^. The ADNI was launched in 2003 as a public-private partnership, led by Principal Investigator Michael W. Weiner, MD. The primary goal of ADNI has been to test whether serial magnetic resonance imaging (MRI), positron emission tomography (PET), other biological markers, and clinical and neuropsychological assessment can be combined to measure the progression of mild cognitive impairment (MCI) and early Alzheimer’s disease (AD).

SuperAgers met previously established criteria (Rogalski et al., 2013). Briefly, they were adults ≥80 years scoring at or above average normative values for adults 50–65 years on an episodic memory test and at least average-for-age in other cognitive domains. This study was carried out in accordance with the recommendations of the Institutional Review Board. The protocol was approved by the Institutional Review Board at Northwestern University and at all of the ADNI sites. All subjects gave written informed consent in accordance with the Declaration of Helsinki.

Controls were adults ≥80 years, scoring within the average-for-age normative range on cognitive tests, including episodic memory.

SuperAging DNA samples were extracted from whole blood using the QIAamp DNA Blood Midi Kits (Qiagen, USA) and analyzed with WES, using the Illumina technology. Libraries were prepared with either the TruSeq 62Mb Exome Enrichment Kit or the Nextera 62Mb Rapid Capture Expanded Exome Kit (Illumina, Inc., San Diego, CA, USA), following manufacturers’ protocols. Exome libraries were sequenced by 100-bp paired-end sequencing on a HiSeq 2500 System (Illumina, Inc., San Diego, CA, USA).

Control WGS data were used to run association analysis and to compare to SuperAger genetic profiles. Control DNA samples were obtained from National Cell Repository for Alzheimer’s Disease (NCRAD) for validation of candidate variants by Sanger sequencing.

Demographic variables were compared using the Wilcoxon-rank sum test or Fisher’s Exact Test. FASTQs were processed with the open-source bcbio-nextgen pipeline^2^, using bwa-mem 0.7.10 for reads alignment, Picard 1.96 to mark duplicates, and GATK Haplotype Caller 3.2 for variants call. The resulting VCF file was filtered to include: Single Nucleotide Variants (SNVs), single nucleotide polymorphisms (SNPs) call rate >85%, Depth >7, and Quality Score >30.

VCF files containing WGS data for controls (*n* = 22) were downloaded from ADNI^3^. We applied the same filters used in the SuperAgers, and excluded SNPs in Hardy-Weinberg disequilibrium (*p* < 1.0E-05). Then, the same SNPs obtained in the SuperAgers were selected after filtering. After combining datasets, a final SNP call rate filter was applied (>95%), and variants were annotated using the “*BumpHunter*” R-package (Jaffe et al., 2012) referencing to the GRCh37/Hg19 assembly.

Population stratification was assessed with the λ inflation factor using the R-package *snpStats* (Clayton, 2015). Association analysis was performed at the gene-level using the Sequence Kernel Association Combined test (SKAT-C; Ionita-Laza et al., 2013) including SNPs located in the promoter and the gene body. Briefly, the SKAT-C test computes a multi-marker statistic assigning the same weight to common and rare variants. Rare variants were defined using the Minimum Allele Frequency (MAF) cutoff computed as MAF = 1/SQRT(2N), where N is total sample size. Analyses using SKAT R-package, accounted for significant covariates. Significant signals were further checked by visual inspection of BAM file reads. *P*-values were Bonferroni corrected. Linkage Disequilibrium between the candidate SNPs was computed using PLINK 1.9 (Purcell et al., 2007).

The frequency of candidate variants was compared with the European (non-Finnish) population data in the ExAC^4^ and the gnomAD databases^5^.

Sanger sequencing of candidate variants was completed on five SuperAgers (selected to include the different genotypes) and 22 Controls. The PCR reactions were performed in 25 μL with AmpliTaq Gold system according to protocol (Applied Biosystems GmbH, Weiterstadt, Germany). Each sample underwent PCR separately for SNPs rs2363221, rs2230435, rs736103 using the primers (5′–3′) Fwd: CTGCTATGAGGCTGGAGTATG+Rev:ACCTCATGCCTTGG GATTT, Fwd:CTGTCATGAGTGTGGGTGTT+Rev:ACCTTCC GTGTCAACTTTAGG and Fwd:TGTCTGCACCAGGATTGT TAG+Rev:CACCTTTCTCAGGACAGAAGTC, respectively. PCR reactions were purified with Agencourt AMPure XP PCR purification (Beckman-Coulter) at a sample/bead ratio of 1:1.8 and sequenced. Samples with ambiguous electropherograms were sequenced twice.

## Results

Table 1 provides group demographics and neuropsychological performance data. The groups were similar in age (*p* = 0.56), but differed in sex (*p* = 8.8E-06) and education level (*p* = 2.8E-03).

**Table 1:** Demographic and neuropsychological performance.

Demographics	SuperAgers^a^	Cognitively average controls^a,b^
	*n* = 56	*n* = 22
Age (years)	83.0 ± 3.3	82.8 ± 2.6
	[80–95]	[79–89]
Education (years)	15.8 ± 2.3	17.7 ± 1.8*
	[12–20]	[14–20]
Sex Men : Women	17:39	19:3*
**Neuropsychological test performance**		
Rey Auditory Verbal Learning Test (RAVLT)	11.6 ± 1.7	4.4 ± 1.4*
Delay Raw Score (out of 15)	[9–15]	[3–7]
Category fluency (animals)	22.8 ± 4.9	19.9 ± 3.6
	[12–33]	[15–27]
Boston Naming Test (BNT; 30-item)	28.4 ± 1.8^b^	28.4 ± 1.7
	[21–30]	[25–30]
Trail Making Test B	88.6 ± 34.5	84.0 ± 32.9
	[38–231]	[41–170]
Mini Mental Status Exam (MMSE)	29.2 ± 1.1	29.0 ± 1.0
	[25–30]	[27–30]

Data are provided as Means ± Standard Deviations and [Ranges]. ^a^Data were from non-Hispanic individuals. ^b^The control genotypes used in the preparation of this article were obtained from the Alzheimer’s Disease Neuroimaging Initiative (ADNI) database (adni.loni.usc.edu). ^b^BNT scores for the SuperAging group were based on n = 55. *Indicates significant differences between the groups. RAVLT: 15-item list learning test of episodic memory with possible scores ranging from 0 to 15. Category fluency: Semantic fluency task in which individuals say as many items from a given category (i.e., animals) as they can in 1 min. BNT: measure of object naming with possible scores ranging from 0 to 30. Trail Making Test B: timed test that assesses executive attention. Test discontinued at 300 s if not completed. MMSE: brief measurement of cognitive functioning with possible scores ranging from 0 to 30.

**Figure 1:**
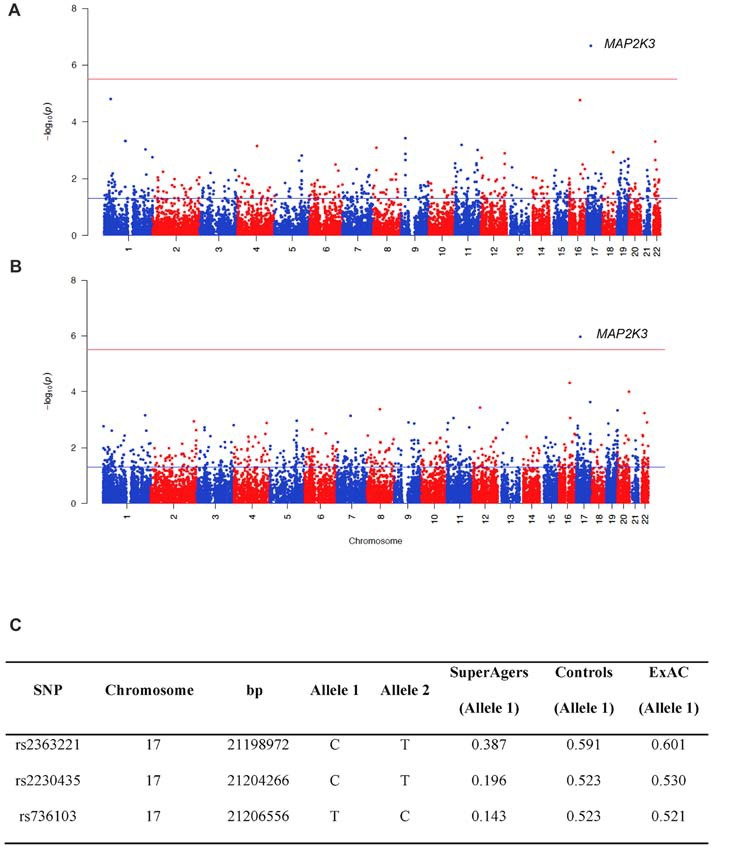
Manhattan plots showing the results of the Sequence Kernel Association Combined (SKAT-C) analysis between SuperAgers and Alzheimer’s disease neuroimaging initiative (ADNI) controls, without covariates **(A)** and with covariates **(B)** included in the model. The red line indicates the significance threshold after Bonferroni correction (*p* = 3.09E-06), whereas the blue line indicates the nominal significance threshold (*p* = 0.05). **(C)** Allele frequencies for the single nucleotidepolymorphisms (SNPs) in the Mitogen-Activated Protein Kinase Kinase 3 (MAP2K3) gene in SuperAging, Control and ExAC cohorts (European non-Finnish population sample).

For WES analysis, after quality controls filtering, there were 145,891 SNPs shared between SuperAgers and Controls. Intergenic SNPs were removed, obtaining 104,731 SNPs in 16,195 genes. Population stratification was not detected (*λ* = 1.013). After removing one false positive by visual inspection of BAM file reads, a significant association for Mitogen-Activated Protein Kinase Kinase 3 *(MAP2K3)* gene was detected in two SKAT-C association analyses conducted with sex and education as covariates and without covariates (*Q* = 37, *p*_adj_ = 0.018; *Q* = 73.4, *p*_adj_ = 0.0011, respectively; Figure 1). Three SNPs contributed to the significance (rs2363221, rs2230435, rs736103) located in intron 1, exon 5 and intron 7, respectively. SNPs rs2230435 and rs736103 showed high Linkage Disequilibrium (*r*^2^ = 0.790), whereas other pairwise *r*^2^ values between SNPs showed *r*^2^ < 0.30 for all comparisons.

SuperAger and Control allele frequencies were also compared with frequencies in the European ExAC cohort and gnomAD database. ExAC and gnomAD cohort allele frequencies were comparable to Controls, but not SuperAgers (Figure 1C).

Using Sanger sequencing, the three variants were successfully validated in 22 ADNI controls and five SuperAgers. The genotype in one sample for the rs2363221 SNP could not be determined.

## Discussion

The SuperAging phenotype was associated with variants in the *MAP2K3* gene. MAP2K3 (or MKK3) is a dual specificity kinase activated by environmental and mitogenic stress residing in a biological pathway linked to memory (Peng et al., 2010) and within a signaling cascade associated with beta-amyloid mediated apoptosis (Zhou et al., 2014). *MAP2K3* is located in chr17:21, 187, 968-21, 218, 552 (GRCh37/hg19) including 11 introns and 12 exons. It is upstream of MAPK14/p38-MAPK, an enzyme altered in AD and a recent therapeutic focus with inhibitors demonstrating positive effects on AD relevant pathology in mouse models (Alam and Scheper, 2016).

MAP2K3 expression is enriched in microglia in mouse cortex (Zhang et al., 2014) and is part of the signaling cascade leading to inflammation (Swaroop et al., 2016), suggesting this variant may be contributing to SuperAging via brain immune system regulation. The role of microglia in AD has recently gained interest due to the association of rare variants in triggering receptor expressed on myeloid cells 2 (TREM2) with significantly altered AD risk (Bellenguez et al., 2017; Sims et al., 2017). The *MAP2K3* gene is significantly upregulated in expression in the middle temporal gyrus of AD patients compared to controls, indicating this gene is regulated to some extent in response to AD.

In view of the small study sample sizes, strict statistical approaches were used to identify the association within *MAP2K3*. Furthermore, data from ADNI were used as controls as these individuals have rigorous phenotype information. We also examined allele frequencies of associated variants in ExAC the largest public human genomic database. Of note, ExAC allele frequencies closely matched our control frequencies. It is possible SuperAger allele frequencies could drift closer to Controls with larger sample sizes; therefore, independent replication is needed.

Our current hypothesis is MAP2K3 activity in the SuperAger brain is slightly decreased due to genetic factors present from birth. None of the identified SuperAger variants are predicted to fully impair MAP2K3 activity. This is notable because MAP2K3 signaling is likely critical for normal cell physiology. This alteration may result in lowered p38-MAPK activity in neuronal cells and may reduce inflammation mediated by microglia (Swaroop et al., 2016), the cell type with the highest known MAP2K3 expression in brain. Further work is necessary to fully explain mechanistic changes and how they are realized in each cell type in SuperAgers. Based on our findings, we postulate MAP2K3 inhibitors may represent a novel therapeutic strategy for enhanced cognition and resistance to AD.

## Author Contributions

Conceptualization of this study was primarily provided by ER, MH, IP, CG and M-MM. Data were collected by ER, EB, SW, EL and M-MM or provided by (ADNI). Data analysis was completed by MH, IP, AS, MD, RR, CB, PJ, EL, M-MM, ER and CG. All authors participated in the writing or revision of this manuscript: MH, IP, AS, MD, RR, CB, PJ, EB, SW, EL, M-MM, CG and ER.

## Conflict of Interest Statement

The authors declare that the research was conducted in the absence of any commercial or financial relationships that could be construed as a potential conflict of interest.

## Footnotes

^1^http://adni.loni.usc.edu/

^2^https://github.com/chapmanb/bcbio-nextgen

^3^http://adni.loni.usc.edu/

^4^ExAC.broadinstitute.org

^5^http://gnomad.broadinstitute.org/
